# Turning *Portunus pelagicus* Shells into Biocompatible Scaffolds for Bone Regeneration

**DOI:** 10.3390/biomedicines12081796

**Published:** 2024-08-07

**Authors:** Louisa Candra Devi, Hendrik Satria Dwi Putra, Nyoman Bayu Wisnu Kencana, Ajiteru Olatunji, Agustina Setiawati

**Affiliations:** 1 Faculty of Pharmacy, Sanata Dharma University, Sleman, Yogyakarta 55281, Indonesia; louisadevi56@gmail.com (L.C.D.); hendriksatria2305@gmail.com (H.S.D.P.); bayuwesn666@gmail.com (N.B.W.K.); 2 CURE 3D, Department of Cardiac Surgery, University Hospital Düsseldorf, 40225 Düsseldorf, Germany; ajiteruolatunji@gmail.com

**Keywords:** polymer, biodegradable, regenerative medicine, bone tissue, crustacean waste

## Abstract

Bone tissue engineering (BTE) provides an alternative for addressing bone defects by integrating cells, a scaffold, and bioactive growth factors to stimulate tissue regeneration and repair, resulting in effective bioengineered tissue. This study focuses on repurposing chitosan from blue swimming crab (*Portunus pelagicus*) shell waste as a composite scaffold combined with HAP and COL I to improve biocompatibility, porosity, swelling, and mechanical properties. The composite scaffold demonstrated nearly 60% porosity with diameters ranging from 100–200 μm with an interconnected network that structurally mimics the extracellular matrix. The swelling ratio of the scaffold was measured at 208.43 ± 14.05%, 248.93 ± 4.32%, 280.01 ± 1.26%, 305.44 ± 20.71%, and 310.03 ± 17.94% at 1, 3, 6, 12, and 24 h, respectively. Thus, the *Portunus pelagicus* scaffold showed significantly lower degradation ratios of 5.64 ± 1.89%, 14.34 ± 8.59%, 19.57 ± 14.23%, and 29.13 ± 9.87% for 1 to 4 weeks, respectively. The scaffold supports osteoblast attachment and proliferation for 7 days. Waste from *Portunus pelagicus* shells has emerged as a prospective source of chitosan with potential application in tissue engineering.

## 1. Introduction

Bone tissue is a rigid tissue that supports other tissue and possesses an excellent capacity for self-healing when it is damaged. However, bone defects and fractures are frequently encountered in orthopedic issues and often necessitate intervention to restore their full function. The prevalence of these conditions is rising, largely due to factors such as trauma, infections, osteoporosis, and tumors, particularly among older adults [1]. While bone tissue has a natural ability to heal and regenerate, allowing minor fractures to mend on their own, more severe cases typically require additional medical treatment [2]. Fractures that exceed a critical size limit (around 2 cm) or result in a significant reduction in bone circumference (more than 50%) may lead to complications like malunion, non-union, and pathological fractures [3,4]. Surgical techniques are generally considered the most effective option for addressing bone defects, despite the variety of treatments available [5]. In orthopedic surgery, bone autografts are widely recognized as the highest standards, as they not only provide a supportive structure for bone healing but also serve as the pool of progenitor cells and crucial biological molecules that promote new bone regeneration [6,7,8]. On the other hand, autografts present some disadvantages, one of which is the necessity of a further procedure which is performed at the site of transplantation. It may cause further issues such as tissue scarring and deformities. They are also costly and associated with risks like bleeding and infection [9]. Additionally, autografts may not be ideal for large defects that require substantial amounts of bone. As a result, there is growing interest in developing alternative bone graft substitutes using biomaterials and tissue engineering to address these challenges and the increasing incidence of bone loss and dysfunction [10,11].

An alternative approach for repairing bone defects is bone tissue engineering (BTE) [12]. The core concept of BTE relies on the integration of cells, a matrix or scaffold, and bioactive growth factors to stimulate tissue regeneration and repair, leading to effective bioengineered tissue [5]. To accelerate bone regeneration, both natural and synthetic polymers are utilized to engineer biocompatible scaffolds. A critical clue of BTE is the development of scaffolds that closely replicate the properties of natural bone [7]. Scaffolds utilized in tissue engineering must meet several key criteria, including the ability to be degraded enzymatically in vivo, less toxicity, suitable surface chemistry, a porous or mesh-like configuration, proper pore size and structure for cell attachment, and the capability to deliver nutrients necessary for cell growth and proliferation [13]. Consequently, a variety of biodegradable scaffolds have been engineered to maintain viable cells in their original state, mimic their natural environments, and promote tissue formation at the transplantation site [14]. These engineered matrix scaffolds act as provisional support for the extracellular matrix (ECM), facilitating cell adhesion, differentiation, and proliferation. Over time, the cells exhibit their natural functions and produce their extracellular matrix, leading to the gradual absorption of the scaffold [12].

Human bone consists of hydroxyapatite (HAP) as the major component alongside other biomaterials like collagen, keratin sulfate, lipids, and chondroitin sulfate [15]. Owing to structural support and biocompatibility, synthetic HAP that is structurally different from natural bone may lead to ectopic bone formation [16]. Collagen type I (COL I) improves HAP’s biocompatibility by facilitating cell adhesion through the engagement of integrin receptor type α1β1 and α2β1 with RGD (arginine–glycine–aspartic acid) peptides [17]. Combining both HAP and COL I in the composite scaffold improves its compatibility with tissues and diminishes toxicity, while the fine particle size of HAP contributes to effective tissue development [18,19]. However, it is a challenging issue to mimic the complex hierarchical structures and dense vascularization of bone tissue. Consequently, chitosan, a naturally occurring biodegradable polymer, has emerged as a very promising biomaterial for scaffold composites in bone tissue regeneration. Its recent popularity is attributed to its ability to be physically and chemically altered, allowing for tailored applications. Key features of chitosan, such as its ability to decompose naturally, compatibility with biological systems, antimicrobial effects, and capacity to form highly porous structures, render it an excellent and adaptable material for tissue engineering [12,14,20,21]. However, chitosan also has some limitations, such as its immunogenicity, weak mechanical properties, and limited availability. To address these issues, research has explored combining chitosan with various other biomaterials [20]. For example, combining chitosan and alginate assists in handling pore size, whereas silica enhances scaffold stiffness and mechanical strength [22,23]. Additionally, the combination of chitosan, collagen I, and hydroxyapatite (HAP) strives to resemble the structure of the bone [24,25].

This study tackles the issue of using chitosan sourced from the waste of blue swimming crab (*Portunus pelagicus*) as a sustainable plentiful resource, in with the Sustainable Development Goals set forth by the United Nations [26,27]. Annual global waste from shrimp, crab, and lobster reaches nearly 6 to 8 million tons, with around 1.5 million tons coming specifically from Southeast Asia [28]. In this study, chitosan was chemically extracted from crab shells and combined with hydroxyapatite (HAP) and collagen type I (COL I) to improve its biocompatibility, mechanical properties, porosity, swelling capacity, and degradation rate. The resulting composite scaffold displayed nearly 60% porosity with pore diameters ranging from 100–200 μm, creating an interconnected network. Consequently, in vitro experiments demonstrated that it supports cell adhesion and the proliferation of osteoblasts. The present work highlights the potential of blue swimming crab (*Portunus pelagicus*) shell debris as an excellent source of chitosan for tissue engineering goals.

## 2. Materials and Methods

### 2.1. Materials

In this study, waste crab shells were collected from Jepara, Central Java. Phosphate-buffered saline (PBS) (10010-023) and bovine collagen I (A10644-01) were supplied from Gibco (Paisley, UK). Commercially available medium molecular weight chitosan and hydroxyapatite were obtained from Sigma (Burlington, MA, USA) (448877 and 677418), and phosphoric acid was purchased from Merck (Darmstadt, Germany) (100573). All tips used in this study were Axygen (Corning, NY, USA), and the e-tube was collected from Eppendorf (Hamburg, Germany). Green Fluorescence Protein (GFP) expressing human dermal fibroblast-neonatal (Angio proteomie, Boston, MA, USA) and MG-63 cells were gifted from prof Kwanwoo Shin, Sogang University, Republic of Korea.

### 2.2. Methods

#### 2.2.1. Preparation of Crab Shells

The University of Gadjah Mada’s Faculty of Biology validated the authenticity of the blue swimming crabs (*Portunus pelagicus*) with authentication number 44/BI/SH/III/2023. The crabs were then meticulously prepared for future usage. After 30 min of boiling in water, the shells were properly rinsed under tap water, allowed to dry for five days, and then blended finely. The powdered shells were kept at room temperature in a jar with silica gel after being sieved through a 100-mesh sieve for optimum preservation.

#### 2.2.2. Chitosan Extraction

The three steps of chitosan extraction are deacetylation, demineralization, and deproteinization. Initially, the sample is deproteinized to eliminate the proteins. Fifteen grams of the sample is combined at a ratio of 1:10 (*w*/*v*) with a 5% NaOH solution. This mixture is cooked for four hours, stirring every five minutes, in a water bath at 80–90 °C. After that, 400 mesh nylon filters are cleaned and filtered through with distilled water to bring the filtrate’s pH down to neutral. The filtrate is then dried for 24 h at 50 °C in an oven. Demineralization is then carried out to get rid of the minerals. The sample is mixed 1:15 (*w*/*v*) with 1.5 N HCl solution. For an hour, the composite solution is continually mixed on a hotplate without being heated. After that, demineralized water is used to wash and filter the filtrate through a 400-mesh nylon filtration until its pH is neutral. The filtrate is dried for 24 h at 50 °C in an oven. The last step is to deacetylate chitin to create chitosan. At a ratio of 1:10 (*w*/*v*), a 50% NaOH solution is introduced to the demineralized sample. For four hours, the mixture is heated in a water bath at 80–90 °C while being stirred every five minutes. A 400-mesh nylon filter is used to cleanse and filter the final filtrate through demineralized water. The following formula is used to determine the chitosan yield:

(1) $$\mathrm{Yield}\;(\%)=\frac{Weight\;of\;Chitosan}{Weight\;of\;crab\;shell\;dry\;powder}\times 100$$

#### 2.2.3. Characterization of Chitosan FTIR

To identify the functional group of chitosan, FTIR spectra were captured using an Agilent system 610 spectrometer [29] with 16 cm^−1^ spectral resolution in the 400–4000 cm^−1^ wavenumber range.

##### Determination of Degree of Deacetylation

In our study, the DD was calculated by the following formula:

(2) $$DD\%=100-\left(\frac{A1320}{A1420}-0.3822\right)\times \frac{1}{0.3133}$$

where A3430 is the absorbance at 3430 cm^−1^ owing to the hydroxyl group as an internal standard and A1655 is the absorbance at 1655 cm^−1^ of the amide I band as a measure of the N-acetyl group concentration. The ratio of this absorbance for an entire acetylated chitosanis 1.33. A suitable baseline was set using Origin software for every spectrum. We compared our isolated chitosan spectra to commercially available chitosan as the control.

#### 2.2.4. Scaffold Fabrication

The scaffold was engineered using techniques from earlier research [19,24]. Initially, 10 mL of 2% (*v*/*v*) acetic acid was used to dissolve 50 mg of chitosan. In the meantime, 10 mL of 2% phosphoric acid (*v*/*v*) was used to dissolve 1.75 g of hydroxyapatite (HAP). Following that, a magnetic stirrer was used for mixing the chitosan and HAP solutions. The mixture was subsequently incorporated with bovine collagen solution in 2% acetic acid (*v*/*v*). Subsequently, a solution of 1 M sodium hydroxide was added to the final chitosan, HAP, and bovine collagen mixture to adjust the pH to 7. After that, the homogeneous liquid was poured into a silicone cast and frozen for a full day at –80 °C. After that, the sample was freeze-dried for an additional 24 h [19]. A further 24 h were used for freeze-drying the sample [19]. Using the same techniques and supplies as the chitosan extracted from blue swimming crab shells, a control scaffold was built for this study using medium-molecular-weight chitosan which was purchased commercially.

#### 2.2.5. Scaffold Morphology Test by Scanning Electron Microscopy

A scanning electron microscope JSM-IT200, JEOL (Tokyo, Japan) was used to observe the chitosan/HAP/collagen scaffold’s microscopic morphology. The scaffold was cut into 2 pieces (vertical and transversal cross-sections), mounted on a disk, and coated with platinum. The SEM then captured images at 50× and 250× magnifications to reveal the pore size and distribution. Thus, energy-dispersive X-ray spectroscopy (EDS) was also employed to evaluate the elemental makeup of the scaffolds.

#### 2.2.6. Porosity Test

The scaffold was fully submerged in anhydrous alcohol and taken out, and any surplus ethanol on its surface was meticulously wiped off. The initial weight of the dried samples was designated as W0, while the weight measured immersion was noted as W1. Three replicates from each category are examined and the scaffold porosity was then determined using the following formula [30].

(3) $$Porosity\;\left(\%\right)\;:\;\frac{W1-W0}{\rho \times V}\times 100\%$$

#### 2.2.7. Compressive Strength Test

Compressive strength was measured with the Tensilon universal testing machine (RTI-1225 A&D Company (Tokyo, Japan)). The identical scaffolds, each measuring 1 cm^3^, were placed into the machine for testing. The experiments were performed at a pressure of 2.5 kN with a rate of 5 mm per minute. The compressive strength of each sample was determined using the following formula:

(4) $$Compressive\;strength\;\left(MPa\right)\;:\;\frac{Fc}{A}$$

#### 2.2.8. Swelling Test

The scaffold was immersed in a phosphate-buffered saline (PBS) solution at 37 °C for 1, 3, 6, 12, and 24 h with three replicates, and the scaffold was removed from PBS. The scaffolds were tapped on filter paper and weighed as W1, while the initial scaffold weight was W0 [23]. The swelling ratio was calculated using the following equation:

(5) $$Swelling\;ratio\;\left(\%\right):\frac{W1-W0}{W0}\times 100\%$$

#### 2.2.9. Degradation Test

The scaffold was immersed in PBS solution with pH 7.4 at 37 °C for 1, 2, 3, and 4 weeks with three replicates. Samples were removed from the PBS solution, oven-dried, and weighed to obtain the final weight as W1, while the initial scaffold weight is W0 [31]. The following equation was employed to calculate the degradation ratio:

(6) $$Degradation\;ratio\;\left(\%\right):\frac{W0-W1}{W0}\times 100\%$$

#### 2.2.10. Cell Biocompatibility Test

GFP-expressing fibroblasts and MG-63 cells were grown in DMEM medium supplemented with 10% fetal bovine serum (FBS) and 1% penicillin-streptomycin. The cell culture was maintained in an incubator at 37 °C a 5% CO_2_ atmosphere. When the cells attained roughly 80% confluency, they were harvested for the experiment. After being rinsed with PBS, they were discarded enzymatically for four minutes in DMEM containing 0.25% trypsin-EDTA. The cells were then collected using an Eppendorf 5810 R centrifuge set to 1000 rpm for three minutes. Subsequently, the collected cells (5 × 10^5^) were seeded on each scaffold which was previously sterilized under UV for an hour. The cells were cultured in a 24-well plate that had been previously coated for 30 min with 5% Pluronic-127. After 24 h, the scaffold was transferred into a newly fresh medium and grown for 7 days. The cell growth was observed under confocal microscopy (Zeiss LSM 500, Carl Zeiss (Oberkochen, Germany)). The fluorescence intensity of the fibroblast represents the quantity of live cells. Additionally, we evaluated cell availability on our scaffold using MG-63, an osteoblast-like cell. A number of 1 × 10^5^ of MG-63 was seeded on each scaffold, similar to the previous method. Twenty-four hours after being transferred into newly fresh media, tetrazolium salt was introduced to the media. The absorbance at 450 nm was measured by formazan salt, which represents the number of living cells. Furthermore, to evaluate cell morphology on the scaffold, the cell-containing scaffold was first treated with 4% paraformaldehyde, followed by ethanol dehydration with 70%, 90%, 96%, and 100% ethanol. After being dried, the scaffold was cross-sectionally cut and observed under an SEM.

#### 2.2.11. Statistics Analysis

The study’s quantitative data is presented as an average value with its associated variability shown by the standard deviation based on at least three separate measurements. Any data points that fell outside the expected range were excluded before further analysis. To compare the data between different groups, one-way ANOVA was used. If a significant difference was found, the Tukey test was used to pinpoint which groups were different from each other. A *p*-value of less than 0.05 was considered statistically significant. The software Origin 9.0 was used to perform these statistical tests.

## 3. Results

**Figure 1:**
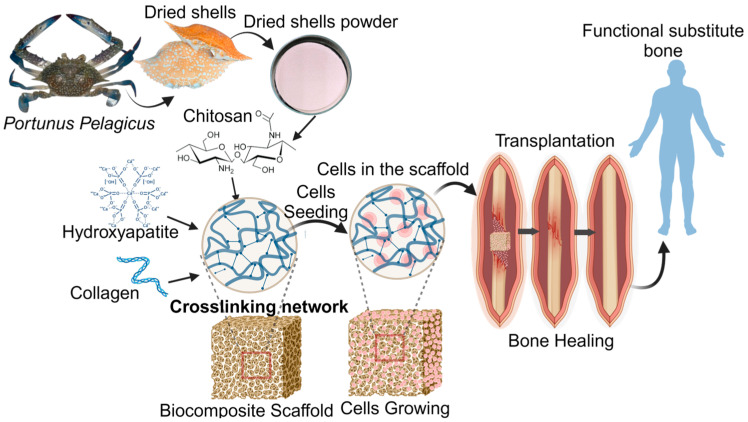
Work scheme of repurposing the shell of Portunus pelagicus for the engineering of bone tissue. This figure was prepared using BioRender.

Recently, natural biodegradable polymers like chitosan have garnered significant interest in the field of tissue engineering. This study focused on obtaining chitosan from the shells of *Portunus pelagicus* to fabricate a composite, which was then fortified with collagen I and hydroxyapatite (HAP) for bone restoration applications. The effectiveness of this composite for bone tissue engineering was assessed by cell seeding on the scaffold, as shown in Figure 1. Chitin, the acetylated precursor of chitosan, is derived from powdered dried crab shells and forms a complex with protein and calcium carbonate. This complex, along with strong hydrogen bonds between adjacent chains, contributes to the low water solubility of crab shells [32,33].

### 3.1. Chitosan Extraction

**Figure 2:**
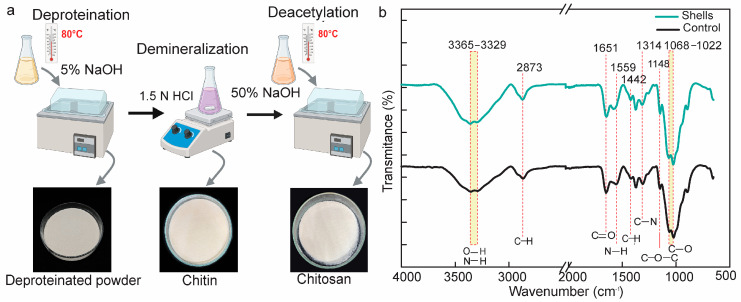
*Portunus pelagicus* shell chitosan extraction and characterization. (**a**) The procedure for extracting chitosan from a *Portunus pelagicus* (PP) shell; (**b**) The chitosan from shell and control’s FTIR spectra (green for shells scaffold and black for control scaffold).

This study isolates chitosan from blue swimming crab (*Portunus pelagicus*) shells in three stages: deproteination, demineralization, and deacetylation (Figure 2a). In the deproteinization stage, proteins are removed by treating the shells with 5% sodium hydroxide at 80 °C, resulting in a protein removal rate of 24.79 ± 8.19%. During the demineralization stage, the remaining shell is treated with 1.5% hydrochloric acid, which dissolves the minerals by reacting with CaCO_3_ and Ca_3_(PO_4_)_2_. The resulting powder is chitin, characterized by poor solubility and reactivity due to its rigid structure and extensive hydrogen bonding. The deacetylation stage involves converting chitin to chitosan by hydrolyzing the acetamide group with concentrated sodium hydroxide, producing acetate ions and an –NH_2_ group, which makes the chitosan acid-soluble. Chitosan, known for its biocompatibility and biodegradability, is broadly utilized in bone tissue engineering. The overall yield of chitosan from *Portunus pelagicus* shells was 9.95 ± 0.92%.

### 3.2. Chitosan Characterization

This study determines chitosan chemical properties based on FTIR spectra. By matching the chitosan fingerprint groups with chitosan as is available commercially, both spectra revealed identical peak patterns. The broad peaks included amine N-H and the hydroxyl O-H stretching peak at 3500 to 3100 cm^−1^ due to overlapping NH and OH groups in both isolated and commercially available chitosan [34]. Both isolated and commercially available chitosan exhibited the stretching band of hydroxyl O-H and amine N-H at 3500 to 3100 cm^−1^ as broad overlapping peaks. Thus, the bands C-H stretching, C=O stretching, and N-H bending in the structure of isolated and commercial chitosan appeared at 2873, 1651, and 1559 cm^−1^ (Figure 2b) [35], while the glycosidic bands, represented by a C–O and a C–O–C bridge, were revealed at 1068–1022 cm^−1^ (Figure 2b) [36,37]. Unlike their monomer counterparts, the glycosidic bonds lead to the emergence of new absorption peak in the 1175–1140 cm^−1^ region (Figure 2b). Differences in the arrangement of glycosidic links are responsible for the unique infrared profile of polysaccharides found in the 1000–920 cm^−1^ region [36]. The specific areas our research confirmed were related to C–C/C–O group stretching vibrations at Region II (1200–800 cm^−1^), OH stretching vibrations at Region V (3600–3050 cm^−1^), and CH/CH_2_ stretching vibration at Region IV (3050–2800 cm^−1^). Therefore, our study effectively extracted chitosan from *Portunus pelagicus* shells that had chemical characteristics that were comparable to those of commercially available chitosan.

Furthermore, we determined the degree of deacetylation (DD), which affects its chemical, biological, and physical properties. The percentage of free amino groups in the polymer chain, which provides the material with a positive charge, affects the degree of deacetylation (DD) of chitosan [38,39]. The study revealed that chitosan DD has an impact on the biocompatibility, biodegradability, hydrophilicity, and adsorption-enhancing properties of biomaterials based on chitosan [40,41]. Compared to commercial chitosan, our previous study determined that the DD of isolated chitosan was 69.89, whereas commercially available chitosan’s DD was 69.34, according to our calculations based on Fatima (2019) [29]. As a result, chitosan from *Portunus pelagicus* shells was successfully separated using our method, and it had chemical characteristics that were comparable to those of chitosan that was sold commercially.

### 3.3. Scaffold Fabrication

**Figure 3:**
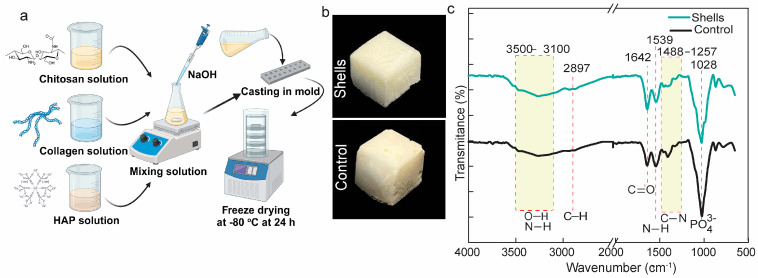
Scaffold composite engineering of extracted chitosan. (**a**) Working design of scaffold composite synthesis; (**b**) Macroscopic profile *Portunus pelagicus’* shell (PP’s shell) and control scaffold; (**c**) FTIR spectra of engineered and control scaffolds (green for shells scaffold and black for control scaffold).

To structurally copy the natural extracellular matrix (ECM) of bone, we created composite scaffolds by combining blue crab chitosan, hydroxyapatite (HAP), and collagen I. This composite was cast into silicon molds and freeze-dried (Figure 3a). Bone composition includes approximately more than 65% calcium phosphate (mainly hydroxyapatite), 21% collagen, 9% water, and 1% other trace constituents. The resulting scaffolds were off-white, with no macroscopic differences between shell chitosan and commercial chitosan (Figure 3b).

### 3.4. Engineered Scaffold Characterization

#### 3.4.1. Scaffold Composition and Microstructure

To verify the fabricated scaffold’s chemical components, we conducted an FTIR experiment. Both scaffolds showed identical peak patterns. Stretching bands for N-H and O-H were observed at 3500–3100 cm^−1^ [42], which correspond to the structures of COL I, HAP, and chitosan. At 1642 and 1488–1257 cm^−1^ [16,43,44], respectively, asymmetric amide I, C=O stretching, and amide III, C–N stretching were found. Furthermore, N-H group bending was identified as the cause of a peak at 1539 cm^−1^ [45]. A previous study revealed that the absorption of the PO_4_^3−^ group is found at 1095, 1031, 961, 603, and 563 cm^−1^ [18]; however, our study only has a clear and strong band characteristic at 1028 cm^−1^, which corresponds to the PO_4_^3−^ group (Figure 3c). Therefore, further investigation is required to precisely identify and comprehend the importance of these extra bands if they exist. EDS was employed as well to assess the scaffolds’ elemental composition. Thus, Figure S1 and Table S1 show that PP’s shell scaffold was composed of O (47.83%), C (21.44%), and Ca (20.36%) while P (10.37%) was one of the minor elements. Strong peaks for phosphate and calcium indicated the presence of HAP in the EDS spectra. Taken together, our method of fabricating the Chi/HAP/COL I scaffold succeeded.

**Figure 4:**
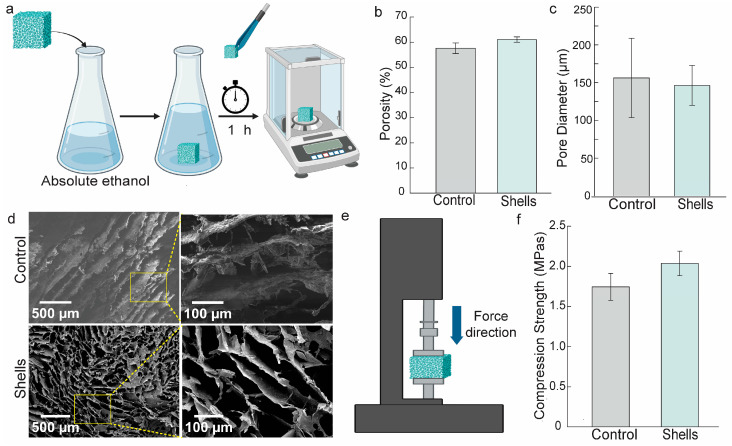
Porosity and microscopic characteristics of engineered scaffolds. (**a**) Work design of porosity measurement method; (**b**) porosity of control and *Portunus pelagicus’* shell (PP’s shell) scaffolds; (**c**) pore diameter of the scaffolds; (**d**) SEM images of control and PP’s shell scaffold; (**e**) scheme of scaffold compressive strength measurement; (**f**) compressive strength of the scaffolds. All quantitative data are given in means ± SD (*n* = 3).

Porosity, pore size, and permeability are a few architectural elements that significantly affect tissue regeneration and biological delivery. One of the most important factors in determining a scaffold’s characteristics is scaffold porosity. Figure 4a shows the scheme of the porosity experiment using liquid displacement methods. Anhydrous ethanol was applied as a liquid displacement as a solvent-free of the scaffold composite, it passed right through the pores without causing any swelling or shrinking [46]. The porosity of the crab shell scaffold was 61.05 ± 1.06%, substantially higher than that of the control scaffold, which had 57.59 ± 2.13% (*p* < 0.05), respectively (Figure 4b).

Furthermore, since the scaffold’s microarchitecture is a key factor in determining its characteristics, we employed scanning electron microscopy (SEM) to observe our scaffold. Based on SEM observation, there was no statistically significant difference between the diameters of the two scaffolds: the control scaffold measured 159.29 ± 52.54 μm, while the chitosan-derived crab scaffold measured 146.31 ± 26.32 μm (Figure 4c). Under SEM analysis, the porosity structure of the crab shell scaffold and the control scaffold was connected and evenly distributed (Figure 4d). Our scaffold consists of interconnecting collagen, HAP, and chitosan, which form a network to help the synthetic tissue transmit waste products and nutrients. The mesoporous structure’s effective interconnection is likely attributed to the amino groups in chitosan forming bonds with collagen and HAP.

Moreover, as mechanical stress is expected for patients during bone regeneration, the bone scaffold should have sufficient mechanical integrity to ensure secure fixation from a biomechanical and therapeutic perspective. The compression test of composite scaffold material was conducted by setting it up in a stationary stage and compressed along its axis by a compressive load during a compression test (Figure 4e). According to Figure 4f, the control scaffold had a compressive strength of 1.74 ± 0.16 MPa, but the PP’s shell scaffold had a value of 2.04 ± 0.15 MPa. Every quantitative data set had three samples and are displayed as means ± SD.

#### 3.4.2. Scaffold Swelling and Biodegradability

**Figure 5:**
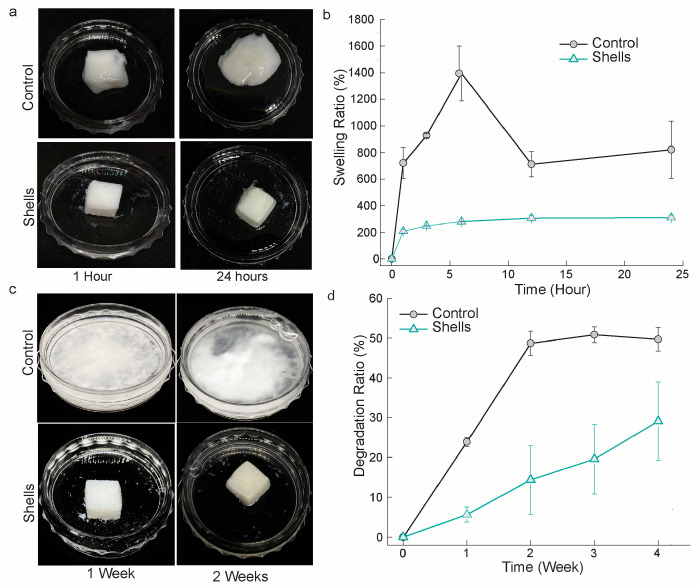
The ratio of swelling and degradation ratio of the fabricated scaffold. (**a**) Swelling scaffold at 1 and 24 h in PBS, (**b**) The ratio of swelling of control and shell scaffolds at 0, 3, 6, 12 and 24 h; (**c**) Degradation morphology of the scaffold at 1- and 2-weeks ratio of the scaffolds, (**d**) The ratio of degradation of of control and shell scaffolds at 1, 2, 3 and 4 weeks. The data are presented as means ± SD (*n* = 4).

The scaffold was submerged in PBS, which mimics biological fluid. After 24 h, the crab scaffold remained structurally robust, but the control scaffold in the PBS had visibly swollen and lost the integrity of its structure (Figure 5a). At 1, 3, 6, 12, and 24 h, the control scaffold’s swelling ratios were 720.73 ± 118.49%, 926.77 ± 14.38%, 1394.84 ± 206.91, 712.00 ± 95.39%, and 820.99 ± 215.56%, respectively. In contrast, the PP shell scaffold showed a noticeably reduced swelling ratio at 1, 3, 6, 12, and 24 h, exhibiting an average of 208.43 ± 14.05%, 248.93 ± 4.32%, 280.01 ± 1.26%, 305.44 ± 20.71%, and 310.03 ± 17.94%, correspondingly (Figure 5b). It was found that our crab scaffolds attained equilibrium swelling by 48 h.

Due to their lower capacity to retain water, our shell scaffold’s decreased swelling ratio may affect its low degradation ratio. It holds its structure until 2 weeks, thus revealing a significant demolished structure at week 4, while the control scaffold disintegrated at week 1 (Figure 5c). At 1, 2, 3, and 4 weeks, the degradation ratios of the control scaffolds were 23.91 ± 1.11%, 48.74 ± 3.08%, 50.88 ± 1.99%, 50.88 ± 1.98%, and 49.75 ± 3.01%, respectively. Conversely, at identical intervals, the shell scaffold’s values were, 19.57 ± 14.23%, and 29.13 ± 9.87%, respectively (Figure 5d).

#### 3.4.3. Scaffold Functionality

**Figure 6:**
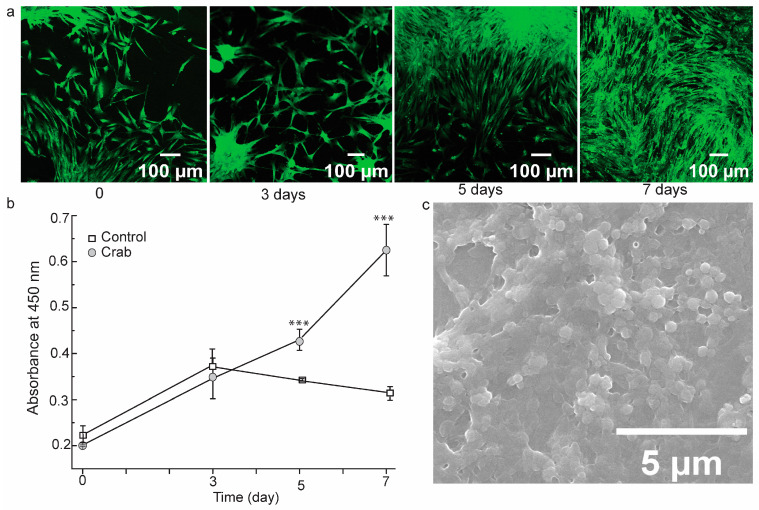
Cell biocompatibility on chitosan derived *Portunus pelagicus*’ shell scaffolds. (**a**) Images of GFP-expressing fibroblasts on the scaffold at 0, 3, 5, and 7 days; (**b**) absorbance at 450 nm after MG-63 cells grown on control and shell scaffold and being treated with tetrazolium salt; (**c**) SEM profile of MG-63 osteoblast-like cell morphology on the scaffold after 7 days. The data are presented in means ± SD (*n* = 3). The statistical significance was computed using a one-way ANOVA followed by the Tukey Test; *** *p* < 0.001.

Since the scaffolds are intended to be used in tissue engineering, it is crucial to investigate their biocompatibility and cytotoxicity. The initial in vitro biofunctionality of the shell scaffolds was further observed using GFP-expressing fibroblast. After seeding the scaffolds with fibroblast, the green fluorescence was highlighted in the living cells, as illustrated in Figure 6a. The cellular density was higher by 3, 5, and 7 days, confirming that the crab scaffold was biocompatible with the cells. Moreover, we investigated osteoblast-like cells’ (MG-63) viability on the scaffold by measuring the absorbance of tetrazolium salt conversion product formazan at 450 nm. At 0, 3, 5, and 7 days, the absorbance values were 0.202 ± 0.00, 0.346 ± 0.04, 0.430 ± 0.02, and 0.625 ± 0.06 (Figure 6b). The scaffold underwent an alteration while observed under an SEM, with MG-63 cells adhering to it and modifying its microstructure from the initial scaffold (Figure 6c).

## 4. Discussion

Biodegradable polymers, or renewable polymers, are highly valued as suitable resource materials due to their exceptional features, including biofunctionality, biodegradability, and non-toxicity [33]. A potential source of the preserved chitosan known as chitin is the blue swimming crab (*Portunus pelagicus*). Chemical extraction is the most widely used procedure in the commercial world, despite being an unfavorable approach that damages the chemical and physical characteristics of chitosan and eliminates minerals and proteins [34,47]. Since the crab shells consist of 25% protein, 25–30% chitin, and 40–50% calcium carbonate [48], it needs three essential steps: deproteination, demineralization, and deacetylation. Some crustacean waste needs decoloration between deproteination and demineralization to remove pigments, such as carotenoid, by treating it with potassium permanganate (KMnO_4_) or hypochlorite [36,37,49]. Since the blue crab shell yielded white demineralized powder, it does not need a decoloration step.

This study fabricated a scaffold to structurally and compositionally mimic bone composition. Bone composition includes approximately 69% calcium phosphate (mainly hydroxyapatite), 21% collagen, 9% water, and 1% other constituents. HAP’s similarity to native bone has sparked interest in bone tissue engineering [50,51], leading to studies exploring HAP-based materials for bone replacement [52,53,54]. Collagen I (COL I), abundant in human tissues (especially bone, where it constitutes over 90% of the ECM) [55,56], acts as a biomimetic integrin-binding peptide (GFOGER) which promotes cell adhesion and differentiation.

Our scaffold is synthesized by a porous and interconnected network between chitosan, HAP, and collagen for transferring nutrients and waste in the newly formed tissue. In tissue engineering, scaffolds have a wide range of porosities, which are frequently customized to meet the unique needs of the intended use. Variations in porosity percentages can occur, ranging from 30% to 90% or higher, depending on several aspects such as the tissue type undergoing regeneration, the intended mechanical characteristics, and the technique employed in the manufacturing of the scaffold. For spongy bone, the range of bone tissue porosity is 70% to 90%, while for compact bone, it is between 5% and 30%. In tissue engineering, porosity is generally customized for each unique application to balance properties like stiffness and cell infiltration; hence, there is no set or typical value for scaffolds [57]. According to research by Lin and colleagues, pores with sizes between 50 and 200 μm offer adequate space for cells to grow and proliferate [58,59]. In the context of bone tissue engineering, this range could extend to as much as 350 μm [60]. This range satisfies the minimal porosity needed for new tissue formation and vascularization by cell proliferation [58].

Additionally, depending on the target bone, the scaffold for bone tissue synthesis must compromise strength. Our scaffold’s strength was far lower than cortical bone’s (which ranges from 100 to 230 MPa) compressive strength, but it was more like cancellous bone’s (2 to 12 MPa) compressive strength [61]. The crosslinking strategy and HAP addition should have improved the scaffold’s mechanical strength. Additionally, as new bone tissue formed into the porous scaffolds after they were implanted in living organisms, their compressive strength increased [62].

Moreover, this study employed an investigation on the swelling ratio of the scaffold as an important characteristic of scaffolds for tissue engineering. Since the swelling ratio in bone tissue engineering may vary depending on various factors, there is not a single, optimal criterion that applies to all conditions. It has been discovered that the water uptake ability of the composite can significantly modify cell proliferation and differentiation and that the scaffold’s ability to retain water is a vital component in determining its eligibility for bone grafting [11]. Furthermore, it has implications for the absorption of water and bodily fluids, as well as for facilitating the movement of nutrients and improving cellular penetration [63]. Water might be absorbed and retained by chitosan scaffolds up to 100% of its weight [62]. The high PBS absorption of the scaffolds is caused by the porous structure of the samples and the hydrophilic groups, such as –OH and –NH_2_ groups [64], in chitosan, collagen, and HAP. For clinical applications, the scaffold’s capacity to biodegrade is extremely advantageous.

In tissue engineering, the scaffold degradation pattern provides an established timetable to produce extracellular matrix and new tissue [61]. The reduced swelling ratio of our scaffold suggests a reduced ability to retain water, which could have an immediate impact on its degradation rate. As a result, this may ultimately lead to the rate of degradation decreasing considerably. Similarly important for preserving the scaffolds’ structural integrity as they encounter biological fluids is controlling the swelling ratio. An optimal scaffold for tissue engineering must have controlled scaffold breakdown [65]. Our scaffold’s lower swelling ratio, which suggests less capacity for water retention, may have a direct impact on the rate at which it degrades. Over time, this may trigger the rate of degradation to slow down. When the scaffold encounters biological fluids, controlling the swelling response is further essential to preserving the scaffold’s structural precision and integrity.

According to prior studies, our scaffold has an appropriate proportion of pores for fibroblast and osteoblast-like cell adhesion and proliferation. Due to this, it supported cell attachment, development, and survival since its composition resembles bone tissue. Our scaffold composition included collagen, which served as the cell binding domain by providing a GFOGFR sequence that binds to cell integrins at α1β1, α2β1, α10β1, and α11β1 [66]. When cells adhere to a biomaterial scaffold, their cytoskeleton responds to the surface of the scaffold through focal adhesion affecting cell behavior, including migration, proliferation, and differentiation. Scaffold design including topographic, geometric, and biochemical cues exhibited a significant impact on cell behavior, including cytoskeletal organization, but not vice versa [67]. Cell–scaffold interactions involve receptor-mediated and mechanical signals, known as mechanotransduction, which governs cell phenotype and function [68]. In the field of tissue engineering for bones, recent studies have used bioactive factors and sensitive factors, altering their properties in real-time [69]. Thus, in vivo research employing animal models is crucial to assess its feasibility for therapeutic applications, especially in non-load-bearing bone lesion types. Consequently, our scaffolds are ideal for in vitro use, and we anticipate that they will continue to be stable when used in vivo.

## 5. Conclusions

In this work, we presented a procedure for isolating chitosan from the shell waste of blue swimming crabs (*Portunus pelagicus*), which was subsequently combined with COL I and HAP. The resulting nanocomposite showed a well-connected network that resembled the architecture of bone tissue extracellular matrix (ECM) and a porous framework with a porosity of 61.05 ± 1.06%. It displayed an ideal scaffold swelling ratio of approximately 800% and a roughly 30% degradation rate throughout four weeks. Our scaffold’s disclosed decreased swelling ratio probably has an impact on how quickly it degrades because of its diminished ability to retain water. For non-load-bearing applications, it matches the compressive strength of cancellous bone quite well, despite having a lower mechanical strength. Through the enhancement of cell proliferation and binding, the collagen addition improved the biological response. Thus, chitosan derived from crab shells offers itself as a viable and sustainable biomaterial for the creation of composite scaffolds in applications involving bone tissue engineering.

## Data Availability

Data are contained within the article and Supplementary Materials.

## Supplementary Materials

The following supporting information can be downloaded at: https://www.mdpi.com/article/10.3390/biomedicines12081796/s1, Figure S1: EDS Analysis of Crab Shell Scaffold; Table S1: Content of Elements by Weight based on EDS spectra.
