# Supplementary material for: Turning Portunus pelagicus Shells into Biocompatible Scaffolds for Bone Regeneration
Source: Biomedicines. 2024 Aug 7;12(8):1796. doi: 10.3390/biomedicines12081796 (PMC11351815; doi:10.3390/biomedicines12081796)
Supplement: Supplementary file 1 [file biomedicines-12-01796-s001.zip › biomedicines-3096051-supplementary.pdf]

Supplementary

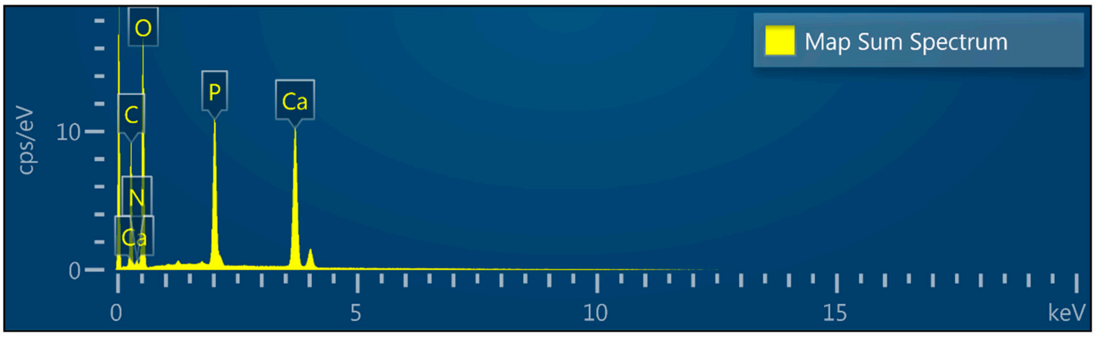

Figure S1. EDS Analysis of Crab Shell Scaffold

Table S1. Content of Elements by Weight based on EDS spectra

Element	Symbol	Weight (%)
Carbon	C	29.95%
Nitrogen	N	3.15%
Oxygen	O	47.03%
Phosphorous	P	6.63%
Calcium	Ca	13.25%
